# Intrinsic motivations and open-ended development in animals, humans, and robots: an overview

**DOI:** 10.3389/fpsyg.2014.00985

**Published:** 2014-09-09

**Authors:** Gianluca Baldassarre, Tom Stafford, Marco Mirolli, Peter Redgrave, Richard M. Ryan, Andrew Barto

**Affiliations:** ^1^Laboratory of Computational Embodied Neuroscience, Institute of Cognitive Sciences and Technologies, National Research CouncilRome, Italy; ^2^Department of Psychology, University of SheffieldSheffield, UK; ^3^Department of Clinical and Social Sciences in Psychology, University of RochesterRiver, New York, USA; ^4^Department of Computer Science, University of Massachusetts AmherstMassachusetts, USA

**Keywords:** intrinsic motivations, novelty and surprise, cumulative learning and development, computational models, autonomous robotics, reinforcement learning, brain and behavior, review

## 1. Introduction

This editorial article introduces the Frontiers Research Topic and Electronic Book (eBook) on *Intrinsic Motivations* (IMs), which involved the publication of 24 articles with the journals *Frontiers in Psychology – Cognitive Science* and *Frontiers in Neurorobotics*. The main objective of this Frontiers Research Topic is to present state-of-the-art research on IMs and open-ended development from an interdisciplinary perspective involving human and animal psychology, neuroscience, and computational perspectives. We first introduce in this section the main themes and concepts on IMs from different interdisciplinary perspectives. These themes and concepts have been reviewed more extensively in other works (e.g., see Barto et al., 2004; Oudeyer and Kaplan, 2007; Mirolli and Baldassarre, 2013; Barto, 2013), but they are briefly reported here both to meet the needs of the reader new to the field and to introduce the concepts and terms we use in the succeeding sections. In the next four sections, we give an overview of the Topic contributions grouped by four themes. A final section draws the conclusions.

Autonomous development and lifelong open-ended learning are hallmarks of intelligence. Higher mammals, and especially humans, engage in activities that do not appear to directly serve the goals of survival, reproduction, or material advantage. Rather, many activities seem to be carried out “for their own sake” (Berlyne, 1966), play being a prime example, but including other activities driven by curiosity and interest in novel stimuli or surprising events. Autonomously setting goals and working to acquire new forms of competence are also examples of activities that often do not confer obvious evolutionary benefit. Activities like these are thus said to be driven by intrinsic motivations (Baldassarre and Mirolli, 2013a). IMs facilitate the cumulative and virtually open-ended acquisition of knowledge and skills that can later be used to accomplish fitness-enhancing goals (Singh et al., 2010; Baldassarre, 2011). IMs continue during adulthood, and they underlie several important human phenomena such as artistic creativity, scientific discovery, and subjective well-being (Ryan and Deci, 2000b; Schmidhuber, 2010).

IMs were proposed within the animal literature to explain aspects of behavior that could not be explained by the dominant theory of motivation postulating that animals work to reduce physiological imbalances (Hull, 1943). The term “intrinsic motivation” was first used to describe a “manipulation drive” hypothesized to explain why rhesus monkeys would engage with mechanical puzzles for long periods of time without receiving extrinsic rewards (Harlow et al., 1950). Other studies showed how animal instrumental actions can be conditioned with the delivery of apparently neutral stimuli: for example, monkeys were trained to perform actions to gain access to a window from which they could observe conspecifics (Butler, 1953), and mice were trained to perform actions that resulted in clicks or in moving the cage platform (Kish, 1955). The psychological literature on IMs initially linked them to the perceptual properties of stimuli, such as their complexity, novel appearance, or surprising features (Berlyne, 1950, 1966). Later, IMs were also related to action, in particular to the competence (“effectance”) that an agent can acquire to willfully make changes in its environment (White, 1959). This relation of IMs with action and their effects was later linked to the possibility of autonomously setting one's own goals (Ryan and Deci, 2000a).

Computational approaches, in particular machine learning and autonomous robotics, are concerned with IMs and open-ended development as these are thought to have the potential to lead to the construction of truly intelligent artificial systems, in particular systems that are capable of improving their own skills and knowledge *autonomously and indefinitely*. The relation of these studies with those on IMs in psychology were first highlighted by Barto et al. (2004) and Singh et al. (2005). The investigation of IMs from a computational perspective can lead to theoretical clarifications, in particular with respect to the computational mechanisms and functions that might underlie IMs (Mirolli and Baldassarre, 2013). IM mechanisms have been classified as being either *knowledge-based* or *competence-based* (Oudeyer and Kaplan, 2007): the former based on measures related to the acquisition of information, and the latter on measures related to the learning of skills. More recently, knowledge-based IMs have been further divided into *novelty-based IMs* and *prediction-based IMs* (Baldassarre and Mirolli, 2013b; Barto et al., 2013). Novelty-based IMs are elicited by the experience of stimuli that are not in the agent's memory (e.g., novel objects, or novel object-object or object-context combinations); prediction-based IMs are related to events that surprise the agent by violating its explicit predictions.

These distinctions have been formalized in the computational models proposed in the literature. Seminal works in machine learning (Schmidhuber, 1991), later developed to function in robots (Oudeyer et al., 2007), have proposed algorithms rewarding actions that allow the agent to improve the quality of a “predictor” component with which it anticipates the effects that such actions produce on the environment. Other researchers have proposed robots capable of detecting and focussing on novel stimuli (e.g., Marsland et al., 2005), or systems capable of detecting anomalies in datasets (Nehmzow et al., 2013). Additional research threads have focussed on action and control, in particular on IMs guiding the autonomous acquisition of motor skills (Barto et al., 2004), on the decision about which of several skills to practice at any time (Schembri et al., 2007; Santucci et al., 2013), and on the the autonomous formation of goals guiding skill acquisition (Baranes and Oudeyer, 2013). Other computational mechanisms related to the idea of IMs are being proposed in the growing field of *active learning*, in particular in relation to supervised learning systems (Settles, 2010).

Recent neuroscientific investigations are revealing brain mechanisms that possibly underlie the IM systems investigated in the behavioral and computational literature. However, unfortunately such investigations are carried out under agendas different from the one on IMs, e.g., in relation to dopamine, memory, motor learning, goal-directed behavior, and conflict monitoring, so comprehensive views are still missing. A large body of research shows how the hippocampus, a brain compound system playing pivotal functions for memory, has the capacity to detect the novelty of various aspects of experience, from the novelty of single items to the novelty of item-item and item-context associations (Ranganath and Rainer, 2003; Kumaran and Maguire, 2007). This detection is then capable of triggering the release of neuromodulators, such as dopamine, that modulate the functioning and learning processes of the hippocampus itself and other brain areas, e.g., of the frontal cortex involved in higher cognition, action planning, and action execution (Lisman and Grace, 2005). Other studies have shown that unexpected stimuli can activate the superior colliculus, a midbrain structure that plays a key role in oculomotor control, which in turn causes phasic bursts of dopamine affecting trial-and-error learning processes happening in basal ganglia, a brain region known to be involved in learning to select actions and other cortex contents (Redgrave and Gurney, 2006). Dopamine signals have also been shown to have an interesting direct relationship with information seeking (Bromberg-Martin and Hikosaka, 2009). Noradrenaline, another neuromodulator targeting a large part of brain, has been shown to be involved in signaling violations of the agent's expectations (Sara, 2009). The failure (Carter et al., 1998) or success (Ribas-Fernandes et al., 2011) in accomplishing goals and sub-goals, possibly themselves set by IMs, has been shown to have neural correlates that might affect succeeding motivation, engagement, and learning. Bio-inspired/bio-constrained computational modeling is linking some of these neuroscientific results to specific computational mechanisms, e.g., in relation to dopamine (e.g., see the pioneering work of Kakade and Dayan, 2002, and Mirolli et al., 2013) and goal-directed behavior (Baldassare et al., 2013).

The 24 interdisciplinary contributions to the present Research Topic can be clustered into four groups. The first group of six contributions (*IMs and brain and behavior*) focuses on different types of IM mechanisms implemented in the brain. The second group of five contributions (*IMs and attention*) focuses on the role of IMs in attention. The third group of eight contributions (*IMs and motor skills*) focuses on IMs as drives for the acquisition of manipulation and navigation skills, often with an emphasis on their function in enabling cumulative, open-ended development. Finally, the fourth group of five contributions (*IMs and social interaction*) focuses on the relationship between IMs and social phenomena, a novel area of investigation of IMs that is increasingly attracting the attention of researchers.

## 2. Intrinsic motivations, brain and behavior

The theoretical contribution of Barto et al. (2013) argues for the importance of distinguishing between *novelty* and *surprise* on the basis of a comprehensive analysis of the computational literature related to the two. It then shows the utility of the distinction for improved understanding of brain and behavior phenomena where the two are often confused. Andringa et al. (2013) present a broad view of possible relationships between IMs and control, exploration, and agency, linking these processes to the specialization of the left and right hemispheres of the brain and showing how the interplay between these can lead to a progressive sophistication of cognition. Shah and Gurney (2014) propose a computational model that investigates how basal ganglia, modulated by IMs, can lead to a dynamical shift from noise-based exploration to repetition that can support the acquisition of both simple and more complex motor skills (in the present case, simulated reaching skills). Boedecker et al. (2013) propose a computational model based on the distinction between dorsal and ventro-medial basal ganglia regions (supporting respectively habitual and goal-directed behavior). Through the model, the authors analyze the relation between these brain regions and IMs concerning reasoning costs and the value of information. This analysis is used to account for some empirical phenomena concerning the relationship between extrinsic and IMs. Fiore et al. (2014) propose a biologically-constrained computational model that also focuses on different portions of basal ganglia. The model shows how these regions can be differentially regulated by a unique tonic dopaminergic signal, linked to both intrinsic and extrinsic motivations, on the basis of their different sensitivity to dopamine. The model, also tested with the simulated humanoid robot iCub, shows how these modulatory mechanisms can play important adaptive functions for the control of overt attention, manipulation, and goal-directed processes. Thirkettle et al. (2013) introduce the novel “Joystick experimental paradigm” developed to study intrinsically and extrinsically driven acquisition of actions. The authors demonstrate the function and effectiveness of this paradigm by presenting behavioral experiments grounded in the neuroscientific literature and concerning the acquisition of non-trivial motor actions.

## 3. Intrinsic motivations and attention

The computational work of Lonini et al. (2013) builds on a previous binocular system in which an IM learning signal is generated on the basis of the capacity of the system to reconstruct images encoded with sparse-coding features. This signal guides the acquisition of attention and vergence skills by reinforcement learning. The contribution here focuses on demonstrating the robustness of the system, in particular for recovering from disturbances and for self-recalibration. Di Nocera et al. (2014) present a behavior-based architecture that uses curiosity drives to improve the attentional capabilities of a reinforcement learning robot engaged in solving simulated survival “extrinsic” tasks. Overall, the work shows the utility of IMs to improve attention and, based on this, action selection. Mather (2013) briefly reviews research related to the familiarity-to-novelty attention shift observed in babies, and, on this basis, highlights the challenges that this phenomenon poses to theories on IMs. Perone and Spencer (2013) also deal with the familiarity-to-novelty shift. In particular, the authors propose a dynamical-field model that offers an explanation of the phenomenon as emerging from the autonomous accumulation of visual experience under the guidance of novelty-based IMs. Schlesinger and Amso (2013), referring to the results of tests of both human and computational agents engaged in solving a visual-exploration task, propose that free viewing of natural images in human infants can be understood as the effect of intrinsically motivated visual exploration driven by the goal of producing predictable gaze sequences. The authors highlight the implications of their approach for understanding visual development in infants.

## 4. Intrinsic motivations and open-ended development of motor skills

Santucci et al. (2013) focus on the problem of which IM signals are best suited to decide which skills to learn by reinforcement learning given a set of tasks. By comparing the results of systems receiving different IM signals, they show that the best IM signals are those based on mechanisms that measure the improvement of the skill competence rather than the errors, or error improvements, of predictors of the action effects on the environment. In a theoretical machine learning contribution, Schmidhuber (2013) proposes a system that automatically invents computational problems in order to train an increasingly-general problem solver. IM signals driving learning are generated when the system finds more efficient skills to solve all the problems generated thus far. In a similar vein, Ngo et al. (2013) propose an architecture for controlling a Katana simulated and real robot interacting with a blocks-world. The system is capable of self-generating goals based on its confidence in its predictions about how the environment will react to its actions. Zahedi et al. (2013) propose the use of task-independent IMs to support task-dependent learning on the basis of the mutual information of the past and future elements of sensor streams (predictive information). The authors conclude that a combination of predictive information with external rewards is recommended only for hard tasks to speed-up learning but at the cost of an asymptotic performance lost. Metzen and Kirchner (2013) propose a reinforcement learning model that self-generates tasks on the basis of graphs of states and selects the skills to learn on the basis of both novelty-based and prediction-based IMs. The system is tested with navigating and octopus-like simulated robots acting in continuous domains. Inspired by infant cognition, Pitti et al. (2013) present a reinforcement-learning bio-inspired gain-fields system for learning task-sets (areas of the sensorimotor space having a common underlying cause-effect structure). The system, tested in a cognitive task and with a Kinova robot arm, is capable of recognizing a given task-set as familiar and can create a new representation for it on the basis of its uncertainty and related prediction errors. Frank et al. (2014) propose a system for controlling the humanoid robot iCub that explores the state-action space on the basis of information gain maximization so as to improve the learning of the world model used for real-time motion planning. Law et al. (2014) present a schema-based memory system inspired by child early sensorimotor development for controlling the iCub robot. The system undergoes a staged learning process to acquire eye-arm reaching skills and basic manipulation skills under the guidance of novelty- and prediction-based IMs, and the progressive release of constraints focussing attention and learning on relevant experiences.

## 5. Intrinsic motivations and social phenomena

In a contribution based on game theory, Merrick and Shafi (2013) propose the concept of “optimally motivating incentive” for game players, and show how different instances of such an incentive (i.e., strong power, affiliation, and achievement motivation) can be used in both modeling human behavior and designing effective artificial agents. The theoretical contribution of Triesch (2013) starts from the idea of IMs serving the function of learning “efficient coding” of sensory data and proposes that imitation can emerge as the consequence of a general intrinsic drive to compress information that leads to matching one's own actions with those of the imitated tutor. Moulin-Frier et al. (2013) propose a model of the initial staged development of speech in infants. IMs initially drive the system to learn the control of phonation, then to produce unarticulated sounds, and finally to produce proto-syllables. The model is tested with a simulator of the vocal tract, the auditory system, the agent's motor control, and social interactions with peers. The contribution of Ogino et al. (2013) proposes a reinforcement learning model of parent-child engagement where learning signals, similar to phasic dopamine signals, are caused by both extrinsic and intrinsic information, in particular related to the presence and novelty of emotional facial expressions. Finally, Jauffret et al. (2013) propose a bio-inspired neural architecture that uses a prediction-based algorithm applied to sensorimotor contingencies to solve complex navigation tasks and is capable of asking for help in dead-lock situations.

## 6. Concluding remarks

The papers of the present Research Topic testify to the existence of ample interest on the Topic issues. At the same time, they show that the literature on IMs is still characterized by a heterogeneity of perspectives on their possible roles in cognition and behavior and on the possible mechanisms supporting them. On the one side, this heterogeneity is expected given the recency of the attempts to systematize the psychological, neuroscientific, and computational views on IMs within broad interdisciplinary frameworks. On the other side, the heterogeneity is also an indication of the richness of intrinsically motivated phenomena, of their importance for animals' cognition and behavior, and of their utility for the design of autonomous robots and intelligent machines. The richness of this topic is expected to result in a further strengthening of the research in the field over the near future.

### Conflict of interest statement

The authors declare that the research was conducted in the absence of any commercial or financial relationships that could be construed as a potential conflict of interest.

## References

[B1] Andringa T. C. van den Bosch K. A. Vlaskamp C. (2013). Learning autonomy in two or three steps: linking open-ended development, authority, and agency to motivation. Front. Psychol. 4:766 10.3389/fpsyg.2013.00766 24155734 PMC3805057

[B2] Baldassare G. Mannella F. Fiore V. G. Redgrave P. Gurney K. Mirolli M. (2013). Intrinsically motivated action-outcome learning and goal-based action recall: a system-level bio-constrained computational model. Neural Netw. 41, 168–187 10.1016/j.neunet.2012.09.015 23098753

[B3] Baldassarre G. (2011). What are intrinsic motivations? a biological perspective, in Proceedings of the International Conference on Development and Learning and Epigenetic Robotics (ICDL-EpiRob-2011), eds Cangelosi A. Triesch J. Fasel I. Rohlfing K. Nori F. Oudeyer P.-Y. (New York, NY:IEEE), E1–E8

[B4] Baldassarre G. Mirolli M. (Eds.). (2013a). Intrinsically Motivated Learning in Natural and Artificial Systems. Berlin: Springer 10.1007/978-3-642-32375-1

[B5] Baldassarre G. Mirolli M. (2013b). Intrinsically motivated learning systems: an overview, in Intrinsically Motivated Learning in Natural and Artificial Systems, eds Baldassarre G. Mirolli M. (Berlin: Springer-Verlag), 1–14 10.1007/978-3-642-32375-1_1

[B6] Baranes A. Oudeyer P. (2013). Active learning of inverse models with intrinsically motivated goal exploration in robots. Robot. Auton. Syst. 61, 49–73 10.1016/j.robot.2012.05.008

[B7] Barto A. (2013). Intrinsic motivation and reinforcement learning, in Intrinsically Motivated Learning in Natural and Artificial Systems, eds Baldassarre G. Mirolli M. (Berlin:Springer-Verlag), 17–47 10.1007/978-3-642-32375-1_2

[B8] Barto A. Mirolli M. Baldassarre G. (2013). Novelty or suprise? Front. Psychol. 4:907 10.3389/fpsyg.2013.00907 24376428 PMC3858647

[B9] Barto A. G. Singh S. Chentanez N. (2004). Intrinsically motivated learning of hierarchical collections of skills, in International Conference on Developmental Learning (ICDL2004), eds Triesch J. Jebara T. (New York, NY:IEEE), 112–119

[B10] Berlyne D. E. (1950). Novelty and curiosity as determinants of exploratory behaviour. Br. J. Psychol. Gen. Sec. 41, 68–80 10.1111/j.2044-8295.1950.tb00262.x

[B11] Berlyne D. E. (1966). Curiosity and exploration. Science 143, 25–33 10.1126/science.153.3731.25 5328120

[B12] Boedecker J. Lampe T. Riedmiller M. (2013). Modeling effects of intrinsic and extrinsic rewards on the competition between striatal learning systems. Front. Psychol. 4:739 10.3389/fpsyg.2013.00739 24137146 PMC3797546

[B13] Bromberg-Martin E. S. Hikosaka O. (2009). Midbrain dopamine neurons signal preference for advance information about upcoming rewards. Neuron 63, 119–126 10.1016/j.neuron.2009.06.009 19607797 PMC2723053

[B14] Butler R. (1953). Discrimination learning by rhesus monkeys to visual-exploration motivation. J. Comp. Physiol. Psychol. 46:95 10.1037/h0061616 13044866

[B15] Carter C. S. Braver T. S. Barch D. M. Botvinick M. M. Noll D. Cohen J. D. (1998). Anterior cingulate cortex, error detection, and the online monitoring of performance. Science 280, 747–749 10.1126/science.280.5364.747 9563953

[B16] Di Nocera D. Finzi A. Rossi S. Staffa M. (2014). The role of intrinsic motivations in attention allocation and shifting. Front. Psychol. 5:273 10.3389/fpsyg.2014.00273 24744746 PMC3978295

[B17] Fiore V. G. Sperati V. Mannella F. Mirolli M. Gurney K. Friston K. (2014). Keep focussing: striatal dopamine multiple functions resolved in a single mechanism tested in a simulated humanoid robot. Front. Psychol. 5:124 10.3389/fpsyg.2014.00124 24600422 PMC3930917

[B18] Frank M. Leitner J. Stollenga M. Forster A. Schmidhuber J. (2014). Curiosity driven reinforcement learning for motion planning on humanoids. Front. Neurorobot. 7:25 10.3389/fnbot.2013.00025 24432001 PMC3881010

[B19] Harlow H. Harlow M. Meyer D. (1950). Learning motivated by a manipulation drive. J. Exp. Psychol. 40:228 10.1037/h0056906 15415520

[B20] Hull C. L. (1943). Principles of Behavior. New York, NY: Appleton-century-crofts

[B21] Jauffret A. Cuperlier N. Tarroux P. Gaussier P. (2013). From self-assessment to frustration, a small step toward autonomy in robotic navigation. Front. Neurorobot. 7:16 10.3389/fnbot.2013.00016 24115931 PMC3792359

[B22] Kakade S. Dayan P. (2002). Dopamine: generalization and bonuses. Neural Netw. 15, 549–559 10.1016/S0893-6080(02)00048-5 12371511

[B23] Kish G. (1955). Learning when the onset of illumination is used as the reinforcing stimulus. J. Comp. Physiol. Psychol. 48, 261–264 10.1037/h0040782 13252153

[B24] Kumaran D. Maguire E. A. (2007). Which computational mechanisms operate in the hippocampus during novelty detection? Hippocampus 17, 735–748 10.1002/hipo.20326 17598148

[B25] Law J. Shaw P. Kevin E. Sheldon M. Lee M. (2014). A psychology based approach for longitudinal development in cognitive robotics. Front. Neurorobot. 8:1 10.3389/fnbot.2014.00001 24478693 PMC3902213

[B26] Lisman J. E. Grace A. A. (2005). The hippocampal-VTA loop: controlling the entry of information into long-term memory. Neuron 46, 703–713 10.1016/j.neuron.2005.05.002 15924857

[B27] Lonini L. Forestier S. Teuliere C. Zhao Y. Shi B. E. Triesch J. (2013). Robust active binocular vision through intrinsically motivated learning. Front. Neurorobot. 7:20 10.3389/fnbot.2013.00020 24223552 PMC3819528

[B28] Marsland S. Nehmzow U. Shapiro J. (2005). On-line novelty detection for autonomous mobile robots. Robot. Auton. Syst. 51, 191–206 10.1016/j.robot.2004.10.006

[B29] Mather E. (2013). Novelty, attention, and challenges for developmental psychology. Front. Psychol. 4:491 10.3389/fpsyg.2013.00491 23914182 PMC3730051

[B30] Merrick K. E. Shafi K. (2013). A game theoretic framework for incentive-based models of intrinsic motivation in artificial systems. Front. Psychol. 4:791 10.3389/fpsyg.2013.00791 24198797 PMC3812661

[B31] Metzen J. H. Kirchner F. (2013). Incremental learning of skill collections based on intrinsic motivation. Front. Neurorobot. 7:11 10.3389/fnbot.2013.00011 23898265 PMC3724168

[B32] Mirolli M. Baldassarre G. (2013). Functions and mechanisms of intrinsic motivations: the knowledge versus competence distinction, in Intrinsically Motivated Learning in Natural and Artificial Systems, eds Baldassarre G. Mirolli M. (Berlin:Springer-Verlag), 49–72

[B33] Mirolli M. Baldassarre G. Santucci V. G. (2013). Phasic dopamine as a prediction error of intrinsic and extrinsic reinforcement driving both action acquisition and reward maximization: a simulated robotic study. Neural Netw. 39, 40–51 10.1016/j.neunet.2012.12.012 23353115

[B34] Moulin-Frier C. Nguyen S. M. Oudeyer P.-Y. (2013). Self-organization of early vocal development in infants and machines: the role of intrinsic motivation. Front. Psychol. 4:1006 10.3389/fpsyg.2013.01006 24474941 PMC3893575

[B35] Nehmzow U. Gatsoulis Y. Kerr E. Condell J. Siddique N. McGinnity M. T. (2013). Novelty detection as an intrinsic motivation for cumulative learning robots, in Intrinsically Motivated Learning in Natural and Artificial Systems, eds Baldassarre G. Mirolli M. (Berlin: Springer-Verlag), 185–207 10.1007/978-3-642-32375-1_8

[B36] Ngo H. Luciw M. Forster A. Schmidhuber J. (2013). Confidence-based progress-driven self-generated goals for skill acquisition in developmental robots. Front. Psychol. 4:833 10.3389/fpsyg.2013.00833 24324448 PMC3840616

[B37] Ogino M. Nishikawa A. Asada M. (2013). A motivation model for interaction between parent and child based on the need for relatedness. Front. Psychol. 4:618 10.3389/fpsyg.2013.00618 24062710 PMC3770941

[B38] Oudeyer P. Kaplan F. (2007). What is intrinsic motivation? A typology of computational approaches. Front. Neurorobot. 1:6 10.3389/neuro.12.006.2007 18958277 PMC2533589

[B39] Oudeyer P. Kaplan F. Hafner V. (2007). Intrinsic motivation systems for autonomous mental development. IEEE Trans. Evol. Comput. 11, 265–286 10.1109/TEVC.2006.890271

[B40] Perone S. Spencer J. P. (2013). Autonomous visual exploration creates developmental change in familiarity and novelty seeking behaviors. Front. Psychol. 4:648 10.3389/fpsyg.2013.00648 24065948 PMC3778377

[B41] Pitti A. Braud R. Mahe S. Quoy M. Gaussier P. (2013). Neural model for learning-to-learn of novel task sets in the motor domain. Front. Psychol. 4:771 10.3389/fpsyg.2013.00771 24155736 PMC3804924

[B42] Ranganath C. Rainer G. (2003). Neural mechanisms for detecting and remembering novel events. Nat. Rev. Neurosci. 4, 193–202 10.1038/nrn1052 12612632

[B43] Redgrave P. Gurney K. (2006). The short-latency dopamine signal: a role in discovering novel actions? Nat. Rev. Neurosci. 7, 967–975 10.1038/nrn2022 17115078

[B44] Ribas-Fernandes J. J. F. Solway A. Diuk C. McGuire J. T. Barto A. G. Niv Y. (2011). A neural signature of hierarchical reinforcement learning. Neuron 71, 370–379 10.1016/j.neuron.2011.05.042 21791294 PMC3145918

[B45] Ryan R. Deci E. (2000a). Intrinsic and extrinsic motivations: Classic definitions and new directions. Contemp. Educ. Psychol. 25, 54–67 10.1006/ceps.1999.1020 10620381

[B46] Ryan R. M. Deci E. L. (2000b). Self-determination theory and the facilitation of intrinsic motivation, social development, and well-being. Am. Psychol. 55, 68–78 10.1037/0003-066X.55.1.68 11392867

[B47] Santucci V. G. Baldassarre G. Mirolli M. (2013). Which is the best intrinsic motivation signal for learning multiple skills? Front. Neurorobot. 7:22 10.3389/fnbot.2013.00022 24273511 PMC3824099

[B48] Sara S. J. (2009). The locus coeruleus and noradrenergic modulation of cognition. Nat. Rev. Neurosci. 10, 211–223 10.1038/nrn2573 19190638

[B49] Schembri M. Mirolli M. Baldassarre G. (2007). Evolving internal reinforcers for an intrinsically motivated reinforcement-learning robot, in Proceedings of the 6th International Conference on Development and Learning, eds Demiris Y. Mareschal D. Scassellati B. Weng J. (New York,NY: IEEE), E1–E6

[B50] Schlesinger M. Amso D. (2013). Image free-viewing as intrinsically-motivated exploration: estimating the learnability of center-of-gaze image samples in infants and adults. Front. Psychol. 4:802 10.3389/fpsyg.2013.00802 24198801 PMC3813899

[B51] Schmidhuber J. (1991). A possibility for implementing curiosity and boredom in model-building neural controllers, in Proceedings of the International Conference on Simulation of Adaptive Behavior: From Animals to Animats, eds Meyer J. A. Wilson S. W. (Cambridge, MA: MIT Press/Bradford Books), 222–227

[B52] Schmidhuber J. (2010). Formal theory of creativity, fun, and intrinsic motivation (1990–2010). IEEE Trans. Auton. Mental Dev. 2, 230–247 10.1109/TAMD.2010.2056368

[B53] Schmidhuber J. (2013). Powerplay: training an increasingly general problem solver by continually searching for the simplest still unsolvable problem. Front. Psychol. 4:313 10.3389/fpsyg.2013.00313 23761771 PMC3675324

[B54] Settles B. (2010). Active Learning Literature Survey. Computer Sciences Technical Report 1648, University of WisconsinMadison

[B55] Shah A. Gurney K. N. (2014). Emergent structured transition from variation to repetition in a biologically-plausible model of learning in basal ganglia. Front. Psychol. 5:91 10.3389/fpsyg.2014.00091 24575067 PMC3920096

[B56] Singh S. Barto A. Chentanez N. (2005). Intrinsically motivated reinforcement learning, in Advances in Neural Information Processing Systems 17: Proceedings of the 2004 Conference, eds Saul L. K. Weiss Y. Bottou L. (Cambridge, MA: The MIT Press).

[B57] Singh S. Lewis R. Barto A. Sorg J. (2010). Intrinsically motivated reinforcement learning: An evolutionary perspective. IEEE Trans. Auton. Mental Dev. 2, 70–82 10.1109/TAMD.2010.2051031

[B58] Thirkettle M. Walton T. Redgrave P. Gurney K. Stafford T. (2013). No learning where to go without first knowing where you're coming from: action discovery is trajectory, not endpoint based. Front. Psychol. 4:638 10.3389/fpsyg.2013.00638 24065941 PMC3776236

[B59] Triesch J. (2013). Imitation learning based on an intrinsic motivation mechanism for efficient coding. Front. Psychol. 4:800 10.3389/fpsyg.2013.00800 24204350 PMC3817656

[B60] White R. W. (1959). Motivation reconsidered: The concept of competence. Psychol. Rev. 66, 297–333 10.1037/h0040934 13844397

[B61] Zahedi K. Martius G. Ay N. (2013). Linear combination of one-step predictive information with an external reward in an episodic policy gradient setting: a critical analysis. Front. Psychol. 4:801 10.3389/fpsyg.2013.00801 24204351 PMC3816314

